# Reassessing Domain Architecture Evolution of Metazoan Proteins: The Contribution of Different Evolutionary Mechanisms

**DOI:** 10.3390/genes2030578

**Published:** 2011-08-05

**Authors:** Alinda Nagy, Laszlo Patthy

**Affiliations:** Institute of Enzymology, Biological Research Center, Hungarian Academy of Sciences, Budapest H-1113, Hungary; E-Mail: nagya@enzim.hu

**Keywords:** epaktologs, evolution of domain architecture, exon-shuffling, gene fusion, gene prediction error, mobility of domains, promiscuity of domains, versatility of domains

## Abstract

In the accompanying papers we have shown that sequence errors of public databases and confusion of paralogs and epaktologs (proteins that are related only through the independent acquisition of the same domain types) significantly distort the picture that emerges from comparison of the domain architecture (DA) of multidomain Metazoan proteins since they introduce a strong bias in favor of terminal over internal DA change. The issue of whether terminal or internal DA changes occur with greater probability has very important implications for the DA evolution of multidomain proteins since gene fusion can add domains only at terminal positions, whereas domain-shuffling is capable of inserting domains both at internal and terminal positions. As a corollary, overestimation of terminal DA changes may be misinterpreted as evidence for a dominant role of gene fusion in DA evolution. In this manuscript we show that in several recent studies of DA evolution of Metazoa the authors used databases that are significantly contaminated with incomplete, abnormal and mispredicted sequences (e.g., UniProtKB/TrEMBL, EnsEMBL) and/or the authors failed to separate paralogs and epaktologs, explaining why these studies concluded that the major mechanism for gains of new domains in metazoan proteins is gene fusion. In contrast with the latter conclusion, our studies on high quality orthologous and paralogous Swiss-Prot sequences confirm that shuffling of mobile domains had a major role in the evolution of multidomain proteins of Metazoa and especially those formed in early vertebrates.

## 1. Introduction

### 1.1. Expected impact of Different Genetic Mechanisms on the Spectrum of Changes in Domain Architecture

#### Unequal crossing-over

It is generally recognized that one of the major genetic mechanisms responsible for changing DA of multidomain proteins is unequal crossing-over that can lead to tandem duplication of domains as well as to deletion of tandem duplicated domains (e.g., ABC ↔ ABBC). All domain-types may be duplicated/deleted by this mechanism in both prokaryotes and eukaryotes but the rate of duplication/deletion may be significantly increased by intronic recombination, explaining why mobile modules (which frequently participate in exon-shuffling) are also prone to undergo tandem duplication [1].

#### Gene fusion

It is widely accepted that fusion of neighboring genes is another major mechanism for DA change in both prokaryotes and eukaryotes. The most plausible pathway for gene fusion in animals is through co-transcription and alternative splicing of neighboring genes, followed by fixation of genomic changes that favor fusion over separate transcription of the constituent genes. This view is supported by the observation that transcripts frequently span two adjacent, tandem genes [2,3]. Typically, such chimeric transcripts begin at the promoter of the upstream gene and end at the termination point of the downstream gene, the intergenic region being spliced out of the transcript by alternative splicing in which the 5' splice site of an intron of the upstream genes is joined to the 3' splice site of an intron of the downstream gene. As pointed out earlier, such cotranscription and intergenic alternative splicing of tandem genes may have played a significant role in the evolution of multidomain proteins of eukaryotes [4].

In the case of gene fusion, the DA of the resulting chimeric gene is dictated by the relative position of the neighboring genes in the species where gene fusion occurs in as much as the upstream gene provides the N-terminal domain(s), whereas the downstream gene provides the C-terminal domain(s) of the chimeric protein. As a corollary, the degree of freedom with which genes (domains) may be combined by gene fusion depends on the rate of genomic rearrangements during evolution.

It must be emphasized that fusion of genes (e.g., when genes with DAs A and B are fused A + B → AB) leads to an N-terminal DA change from the perspective of the downstream gene (gene with DA = B; B→ AB) and C-terminal change from the perspective of the upstream gene (gene with DA = A; A→ AB), therefore the frequencies of N-terminal and C-terminal DA changes due to gene fusion are expected to be similar.

#### Gene fission

Most studies agree that the process when a gene encoding a multidomain protein is ‘split’ in a way such that its constituent domains are expressed separately (AB → A + B) is much rarer than gene fusion. [5,6]. A plausible explanation for the infrequency of gene fission relative to gene fusion is that it is much easier to lose the 5' termination signal of the upstream gene and the *cis* regulatory regions of the downstream gene (as in gene fusion, see above) than to gain both of these (as in gene fission). Since the balance of gene fusion/fission is thus tilted in favor of fusion, it is frequently assumed that gene fusion is the main driver of the evolution of more complex multidomain proteins.

We wish to emphasize that fission of genes (resulting in DA change of the type AB → A + B) involves an N-terminal change from the perspective of one of the resulting genes (gene with DA=B; AB → B) and C-terminal change from the perspective of the other gene (gene with DA =A; AB → A), therefore the frequencies of N-terminal and C-terminal DA changes due to gene fission are also expected to be similar. It should be noted, however, that the term gene fission is sometimes (e.g., [7]) used in a different sense: loss of terminal domains (e.g., AB → A or AB → B).

#### Point mutations changing the boundaries of the Open Reading Frame

In principle, changing signals for translation initiation and termination may favor loss over gain of terminal domains, since the use of a novel translation initiation site upstream of the original site (converting 5'-untranslated region to translated region) or conversion of the original stop codon to a sense codon (converting the 3'-untranslated region to translated region) is unlikely to result in the gain of a new domain since the 5'- and 3'-untranslated regions were not selected to encode folded domains. Conversely, the gain of a novel translation initiation site downstream of the original one may lead to the loss of an N-terminal domain (e.g., AB → B) or the gain of a novel translation termination site upstream of the original one may lead to the loss of C-terminal domains of a protein (e.g., AB → A). As a corollary, changing signals for translation initation and termination would favor loss over gain of terminal domains. As to the relative probabilities of loss of N-terminal or C-terminal domains: we must take into account the fact that nonsense-mediated decay (NMD) may detect premature stop codons [8] and may prevent the formation of C-terminally truncated proteins and thus act against the loss of C-terminal domains.

#### Exon-shuffling and other ways of domain-shuffling

It is generally agreed that shuffling of symmetrical class 1-1 domains (domains flanked by phase 1 introns) by intronic recombination contributed significantly to the evolution of multidomain proteins of Metazoa but it is also clear that intronic recombination is not an absolute prerequisite of domain-shuffling [1,9–14].

Analysis of a large number of cases where the evolutionary history of the DA change involving class 1-1 domains could be reliably reconstructed revealed that—as a rule—exons/exons-sets encoding class 1-1 domains are inserted in pre-existing phase 1 introns of the recipient gene [13]. The resulting DA change may be classified as N-terminal, C-terminal or internal DA change, depending on the position of the intron where the class 1-1 domain was inserted. For example, in the collection of examples discussed in the accompanying papers the class 1-1 TSP1 domains were inserted internally in the case of the thrombospondin family (see TSP2_HUMAN), the class 1-1 Laminin EGF domains and SEA-domains were inserted internally during evolution of agrins [15], the class 1-1 FN1 domain was inserted at an N-terminal position during evolution of plasminogen activators (see TPA_HUMAN; [8]), the class 1-1 kringle-domain was inserted internally in thrombin during evolution of blood coagulation proteins (see THRB_HUMAN; [8]), the class 1-1 FN2 domain was inserted internally during evolution of the MMP and SE1L families (see MMP2_HUMAN and SE1L1_HUMAN).

The same genomic features that are essential for exon-shuffling (introns of identical phase at the boundaries of the domain that is shuffled) also facilitate the loss of domains acquired by exon-shuffling through fixation of exon skipping [16].

A survey of the various genetic mechanisms that may change the DA of proteins thus suggests that unequal crossing-over, gene-fusion and domain-shuffling are the ‘creative’ mechanisms that may increase the complexity of the DA of multidomain proteins. Conversely, the DA complexity of multidomain proteins may be decreased by unequal crossing-over, gene fission, point mutations that change the boundaries of the open reading frame and fixation of exon-skipping.

It must be emphasized that a major difference between gene fusion and domain-shuffling is that the former may alter DAs only at the termini (e.g., A + B → AB or AB +C → ABC or A +BC → ABC) whereas exon-shuffling does not have this requirement: it may add domains both internally (e.g., AB + C → ACB) or at the termini (e.g., AB + C → ABC or AB + C → CAB). As a corollary, the relative frequency of DA change in internal positions versus N-terminal and C-terminal positions may be used to assess the relative contribution of gene fusion and domain-shuffling to DA evolution.

For example, if we assume that gene fusion is the dominant mechanism responsible for DA change we expect that the rates of DA change at the N-terminal and C-terminal ends of proteins significantly exceeds that observed at internal positions. Conversely, if we assume that domain-shuffling was the dominant mechanism responsible for DA change we expect that the rates of DA change at N-terminal, C-terminal and internal positions are roughly similar. It should be noted, however, that this analysis would be meaningless if we disregard the fact that in the case of one-domain ↔ two domain transitions (type 1 transitions that account for the majority of DA changes), domain architecture change can only be classified as terminal (e.g., A ↔ AB or A ↔ BA).

### 1.2. Expected impact of Different Genetic Mechanisms on the Versatility, Promiscuity and Mobility of Domains

Domains of multidomains proteins are frequently labeled with epithets to express some aspects of their role in the evolution of different domain architectures. The most frequently used terms are ‘versatile domains’, ‘promiscuous domains’, ‘mobile domains’ and sometimes these terms are used as if they were synonyms [17].

The generally accepted definition of domain promiscuity/versatility is that domains are promiscuous/versatile if they are present in many different domain architectures [18–20]. Typically, the degree of promiscuity of a domain is defined as the number of distinct architectures in which it is present or the number of domain-types associated with it. For example, the Pfam A domains ‘trypsin’ and ‘pkinase’ have high promiscuity/versatility scores since they combine with a large variety of domains to form a rich repertoire of domain architectures. It should be noted, however, that the terms promiscuity/versatility have no implications as to the genetic mechanism that alters DA.

In contrast with this, the term ‘domain mobility’ is intended to reflect the frequency with which a domain is shuffled, *i.e.*, moved from one local environment (within a gene) to a new local environment (of another gene). Accordingly, the mobility of a domain is related to but not equivalent with its versatility/promiscuity: mobile domains are necessarily versatile, but versatile domains are not necessarily mobile. For example, domain A is involved in a mobility event if it is inserted into a new environment of a recipient gene (e.g., encoding a protein with DA XYZ to give protein with DA of XYAZ) but the XY and Z domains of the recipient gene are not since they did not move from their original environment: the mobility score of domain A is increased by one count, but the mobility scores of X, Y and Z are unaffected. The situation is quite different for versatility/promiscuity scores: the DA change of XYZ → XYAZ will equally affect the versatility/promiscuity score of all four domains involved. This point may be illustrated by the cases of the trypsin domains of regulatory proteases such as TPA_HUMAN, THRB_HUMAN or NTR_HUMAN: these vertebrate-specific multidomain architectures arose by shuffling of mobile domains (e.g., class 1-1 kringle-, FN1-, SRCR-domains) whereas, in these DA changes, their common trypsin domain served as recipients for the mobile domains.

Despite the practical problems associated with genome-scale reconstruction of such events some prototypical studies may serve to illustrate the importance of the distinction of versatility and mobility of domains. Analysis of the evolutionary history of proteases of the blood coagulation and fibrinolytic cascade revealed that during evolution of these paralogous proteins of the trypsin-family a variety of mobile modules (e.g., kringle-, EGF-, FN1- and FN2-modules) were inserted into the ‘recipient’ genes [9,21]. In terms of versatility, each acquisition of a novel mobile module increases the versatility/promiscuity score of the trypsin-like protease domain of the recipient genes, even though its mobility score is unaffected.

In our definition domain-shuffling (domain mobility) is restricted to cases where the partners involved in DA changes are non-equivalent; the domain is moved from one genome location (a donor gene) to a recipient gene. This definition excludes gene fusion as a mobility event since both partners can be considered donor and acceptor. Conversely, domain insertion (e.g., by exon-shuffling) is a mobility event; since the roles of the recipient gene and donor genes are non-equivalent and can be clearly distinguished.

### 1.3. Expected Impact of Sequence Errors on Conclusions About Domain Architecture Evolution of Metazoan Proteins

As emphasized in an accompanying paper (Nagy, Szláma, Szarka, Trexler, Bányai, Patthy, Reassessing Domain Architecture Evolution of Metazoan Proteins: Major Impact of Gene Prediction Errors), reliable analysis of DA evolution of multidomain proteins requires that the protein sequences compared are valid, correct and complete. It must be pointed out that many authors realized that gene annotation errors may cause some problems in the analysis of DA of proteins but most studies implicitly assumed that such errors may be neglected in genome-scale analyses and that they occur at random thus they do not obscure the general tendencies of DA evolution. We have shown in an accompanying paper that neither of these assumptions is justified (Nagy, Szláma, Szarka, Trexler, Bányai, Patthy, Reassessing Domain Architecture Evolution of Metazoan Proteins: Major Impact of Gene Prediction Errors). First, in the case of most Metazoan species the contribution of gene prediction errors to domain architecture differences of orthologous and paralogous proteins is comparable/greater than those of true gene rearrangements. Second, the accuracy of gene prediction itself has a strong positional bias, in as much as it is most reliable for internal exons and least reliable for N-terminal exons [22], thus, errors in gene prediction do not merely increase the rate of DA differences at random: they introduce a strong positional bias in favor of apparent terminal DA changes.

As discussed above, neither unequal crossing-over, nor gene fusion nor domain-shuffling are expected to favor N-terminal versus C-terminal DA change, thus we have no obvious genetic explanation for the observation in genome-scale studies that DA changes are preferred at the N-terminal end [5]. It seemed more likely that this observation reflects the fact that the majority of erroneous (incomplete, mispredicted) sequences present in databases such as TrEMBL, EnsEMBL differ from the correct sequence more frequently at the N-terminal end than the C-terminal end. By analyzing type 1 transitions (one domain ↔ two domain transitions), type 2 transitions, (two-domain ↔ three domain transitions) and type 3 transitions (N-domain ↔ N + 1-domain transitions, where N is greater than 2), separately we have shown that in the case of high quality Swiss-Prot proteins of Metazoa the probability of DA change is similar at internal and terminal positions. In contrast with this, in the case of TrEMBL (where a significant proportion of the sequences is incomplete, incorrect or aberrant), RefSeq, EnsEMBL and NCBI's GNOMON predicted sequences (that frequently contain mispredicted sequences) the apparent rate of terminal changes were significantly increased relative to internal changes.

Our findings thus cautioned that earlier proteome-scale studies that neglected the contribution of sequence errors may have led to erroneous conclusions about the evolution of novel domain architectures of multidomain proteins. Our observation on high quality Swiss-Prot sequences, that the contribution of internal DA alterations increased in vertebrates (Nagy, Banyai and Patthy, Reassessing Domain Architecture Evolution of Metazoan Proteins: Major Impact of Errors Caused by Confusing Paralogs and Epaktologs), is consistent with our suggestions that exon-shuffling played a major role in shaping the DA of multidomain proteins unique to vertebrates [12].

### 1.4. Expected Impact of Confusing Epaktologs and Paralogs on Conclusions about Domain Architecture Evolution of Metazoan Proteins

We have demonstrated that contamination of protein families with epaktologs (proteins that are related only through the independent acquisition of the same domain types) increases the apparent rate of DA change and introduces a strong bias in DA differences in as much as it increases the proportion of terminal over internal DA differences (Nagy, Banyai, Patthy, Reassessing Domain Architecture Evolution of Metazoan Proteins: Major Impact of Errors Caused by Confusing Paralogs and Epaktologs). These findings cautioned that earlier studies based on analysis of datasets of protein families that were contaminated with epaktologs may have led to some erroneous conclusions about the evolution of novel domain architectures of multidomain proteins.

## 2. Results and Discussion

In view of our observation that sequence errors and confusion of epaktologs with other types of homologs significantly distorts the evolutionary history of the DA of multidomain proteins, it is important to re-examine the conclusions of earlier studies that neglected the influence of these errors. As emphasized in the accompanying papers, these errors not only increase the apparent rate of DA change but they also introduce a strong positional bias in favor of terminal over internal DA changes.

In the case of bacterial genomes (where it is justified to neglect misprediction) it was convincingly shown that terminal changes are significantly (more than 10-fold) more frequent than internal ones [23]. Interestingly, studies that analyzed datasets of Archean, Bacterial, Eukaryotic proteins have noted a similar degree of bias in favor of terminal over internal DA changes for eukaryotes and prokaryotes, leading several authors to conclude that this bias is also valid for eukaryotic organisms [5,6,24–27]. Since there are major differences in the organization of genomes/genes of prokaryotes and higher eukaryotes such as Metazoa one would expect that these differences have some impact on DA evolution. Indeed, there is a general consensus that the rate of formation of new DAs is significantly higher in Metazoa than in prokaryotes or other eukaryotes [25] so it is even more surprising that this increase in the rate of DA evolution (that is generally attributed to an increased role of exon-shuffling in the Metazoan lineage [12]) is not reflected in a shift in favor of internal DA changes.

We suggest that the absence of this shift is due to the fact that in most studies the high proportion of incomplete, abnormal or mispredicted sequences of higher eukaryotes increased the rate of terminal *vs.* internal changes and this was taken as evidence for gene fusion. This point may be illustrated by the work of Weiner *et al.* [24]. These authors have analyzed the whole SwissProt/TrEMBL set of proteins and concluded that DA changes occur most frequently at termini which in turn led the authors to conclude that “these results have further supported the emerging view that, by and large, the modular evolution of proteins is dominated by two major types of events: fusion, on the one hand, and deletion and fission on the other”. Buljan and Bateman [27] have also studied domain architecture evolution in animal gene families represented in UniProt (Swiss-Prot plus TrEMBL) database and these authors have also concluded that gain and loss of domains is preferred at protein termini. As we have pointed out in an accompanying paper (Nagy, Szláma, Szarka, Trexler, Bányai, Patthy, Reassessing Domain Architecture Evolution of Metazoan Proteins: Major Impact of Gene Prediction Errors), as a consequence of the large proportion of incomplete sequences in the TrEMBL section of UniProtKB the DA of these erroneous sequences differ from those of the correct sequences at the termini thus falsifying the positional distribution of DA changes during protein evolution. Accordingly, the conclusions drawn from DA analysis of datasets dominated by TrEMBL sequences seem to be unjustified.

In their genome-scale studies on DA evolution, Ekman *et al.* [25] have used the EnsEMBL proteomes for the eukaryotic genomes. They also concluded that most events involve a single domain, which is inserted at the N or C termini, implying that gene fusion is the dominant mechanism for DA change. As we have pointed out, as a consequence of the large proportion of mispredicted sequences in the EnsEMBL proteomes the DAs of these erroneous sequences differ from those of the correct sequences at the termini thus falsifying the positional distribution of DA changes during protein evolution. Accordingly, the conclusions drawn from DA analysis of EnsEMBL sequences seem to be unjustified.

Despite the fact that the use of erroneous sequences and other types of methodological errors casts doubt on the results of such analyses the view that the major genetic operations leading to novel arrangements are fusion of existing genes and terminal loss of domains is gaining popularity since it is propagated in several recent review papers [26,28].

Realizing the danger of confusing gene- and protein-annotation errors with true changes of DA, in a recent paper Buljan *et al.* [29] have chosen the strategy that instead of genome-scale analysis of DA evolution of datasets (of dubious quality) they analyzed a limited set of cases that they considered as high confidence domain gain events in Metazoa. Based on these studies they have reached the same conclusion as in their earlier genome-scale studies: “the major mechanism for gains of new domains in metazoan proteins is likely to be gene fusion through joining of exons from adjacent genes”. The authors have also concluded that “insertion of exons into ancestral introns through intronic recombination are, in contrast to previous expectations, only minor contributors to domain gains” and have accounted for less than 10% of high confidence domain gain events. Buljan *et al.* also noted that the DA change occurs more frequently at the N-terminal than the C-terminal end. In a Research Highlight commentary of the work of Buljan *et al.* Marsh and Teichmann [30] have concluded that “although recombination between introns has been speculated to be one of the main mechanisms behind the diverse domain rearrangements observed in complex eukaryotes, it seems to have made a fairly limited contribution to the domain gain events”.

Since these conclusions contradict our data and the widely accepted view that exon-shuffling played a major role in DA evolution of proteins unique to Metazoa we have carefully examined the evidence presented in the paper of Buljan *et al.* [29]. Our analyses identified four major types of problems with the analysis of Buljan *et al.* that explain this contradiction.

The first and most important problem is that the set of ‘high-confidence domain gain events’ does not properly represent the spectrum of DA changes (e.g., noteworthy absence of numerous, well documented, high-confidence domain gain events). The authors acknowledge that the limited set (330 cases) of ‘high-confidence domain gain events’ may not properly represent the whole spectrum of DA changes: “even though we do not expect that the final set of high-confidence domain gains is biased towards any of the mechanisms, the total number of gain events in the set is relatively small and this could introduce apparent dominance of one mechanism over another”. To check this possibility they use a larger number of ‘medium-confidence’ domain gains (849 cases) to “test whether a larger set of domain gains would support the observed distribution of characteristics of gained domains”. Although they note that the “major difference between the two sets was in the number of middle domains coded by one exon” they dismiss this warning sign (pointing to the role of exon-shuffling) by saying that “we believe that this is largely due to false domain gain”.

The problems with the data set of Buljan *et al.* are probably best illustrated by the fact that many of the best-known, well-documented cases of domain-gains are missing from the list (Table S1 in [29]). If we check the presence/absence of the examples discussed in the accompanying papers we find that the majority are missing. For example, TreeFam tree TF329901 that presents many well-documented, exon-shuffling mediated cases of DA rearrangements of plasminogen-related proteins, urokinase, tissue-plasminogen activator (see TPA_HUMAN), hyaluron-binding protein, blood coagulation factor 12, hepatocyte-growth factor activator etc. is missing from the list of high-confidence domain gain events. Similarly, trees TF315428, (containing MMP2/MMP9, the common ancestor of which is known to have gained FN2 domains by exon-shuffling; see MMP2_HUMAN), TF324917 (containing TSP1/TSP2 (the common ancestor of which is known to have gained TSP1 domains by exon-shuffling; see TSP2_HUMAN), TF315257 (containing sel-1 homologs where SE1L1 proteins are known to have gained an FN2 domain by exon-shuffling; see SE1L1_HUMAN; TF326548 (containing agrins that are known to have gained a SEA domain by exon-shuffling in the chordate lineage; see AGRIN_HUMAN), TF317274 (containing amyloid A4 precursor, known to have gained a Kunitz domain in the vertebrate lineage by exon-shuffling, see A4_HUMAN) are also missing from the list of ‘high-confidence domain gain events’.

Another important problem with the analysis of Buljan *et al.* is that the conclusions are sometimes erroneous as a consequence of the fact that TreeFam sometimes confuses paralogs and epaktologs. As discussed in an accompanying paper (Nagy, Banyai and Patthy, Reassessing Domain Architecture Evolution of Metazoan Proteins: Major Impact of Errors Caused by Confusing Paralogs and Epaktologs), this type of error is encountered most frequently in the case of epaktologous proteins that contain tandem repeats of the same domain-type. For example, in tree TF326157 (Family Name: Complement factor H-related protein precursor) complement factor H and factor XIIIb that arose in vertebrates (consisting of tandem sushi domains) are claimed to belong to the same family as some invertebrate proteins (e.g., CG10186, Q8INW2_DROME) simply because they also contain multiple tandem sushi domains.

As a consequence of the contamination of some TreeFam trees with epaktologs the evolutionary history of the DA of proteins and the conclusions drawn from this history may be erroneous. The case of TF329295 is especially instructive. The common feature of proteins listed in this tree is that they contain tandem SRCR domains, including CD5-, CD6-, SRCRL-, C163A-, DMBT1- and NETR-related sequences, thus the tree implicitly assumes that they are all descendants of a common ancestor with multiple SRCR domains. Consequently, the tree implies that additional domains that are present only on some branches of the tree (zona_pellucida domain in DMBT1-related sequences and trypsin in NETR-related sequences) (see Figure 2/c of [31]) were gained in the corresponding trees. As a consequence, in the list of ‘High confidence domain gain events’ (Table S1 of [29]) the authors claim that in the TF329295 family a trypsin domain (CL0124 Peptidase_PA, trypsin) has been gained in vertebrates. As representative transcript with the gained domain they give ENST00000296498 (protein ID ENSP00000296498 equivalent with neurotrypsin, NETR_HUMAN). The basis of this interpretation is that the tree TF329295 implies that the closest paralogs of neurotrypsin are CD5-, CD6-, DMBT-, WC11-related proteins *etc.* that all contain multiple SRCR domains. In contrast with this interpretation, neurotrypsins are paralogs of the plasminogen-activator branch of trypsin-like proteins (based on the evolutionary affiliations of its kringle and protease domains) and the present DA of neurotrypsin evolved through the gain of internal mobile SRCR domains by an ancestral protease and not through the gain of a terminal trypsin domain by an ancestral CD5-like protein. Similarly, in the list of ‘High confidence domain gain events’ in TreeFam family tree TF329295 ENSMUST00000084509 (ENSMUSP00000081556, corresponding to DMBT1_MOUSE) is listed as an example of domain gain, claiming that this protein arose from an ancestor with multiple SRCR domains by gaining a terminal PF00100 (Zona_pellucida) domain, rather than from an ancestor with a Zona_pellucida domain through acquisition of mobile SRCR domains.

A third major source of errors is that the authors rely on EnsEMBL sequences, therefore the analysis inherits the problems (presence of erroneous sequences) of this database. For example, when we analyzed the dataset by blasting the EnsEMBL entries against UniProtKB we identified several cases where the corresponding UniProtKB entry was annotated as ‘no protein product’ or ‘retired entry’.

Sometimes the authors draw conclusions based on analysis of abnormal transcripts. In Additional file 8 of [29] the authors illustrate their conclusions by highlighting some examples of domain gains by joining of exons from adjacent genes. Here the authors discuss the case of CELSR3 (Treefam tree TF323983) containing Cadherin EGF LAG seven-pass G-type receptor (CESLR) precursor genes as an example of DA change in the vertebrate lineage. The authors conclude that “one branch of the family, containing vertebrate genes, has gained the Sulfate transport and STAS domains in addition to the ancestral cadherin, EGF and other extracellular domains” and suggest that “the gain occurred after the other vertebrates diverged from fish and homologues without the gained domains are present in all animals.” A closer examination of this case, however, questions the validity of the conclusion that a gene fusion has occurred. The single experimental evidence supporting this claim is that a cDNA (Accession AY714129) was cloned that arose as a result of co-transcription of the closely linked genes for the cadherin EGF LAG seven-pass G-type receptor 3-like protein CELR3_HUMAN and the gene for sulfate transport protein S26A6_HUMAN. When we searched the human EST database with the ‘fusion’ region we identified numerous ESTs for the separate transcription of the two genes but no EST supporting the fusion. These results indicate that a rare event of co-transcription and alternative splicing gave rise to transcript AY714129 but this should not be confused with genomic rearrangement leading to DA change. It is worth noting that the transcript AY714129 is also abnormal in the sense that the protein product (Accession AAU94938) lacks the cleavable signal peptide of CELR3_HUMAN, so the putative protein (Q5Y190_HUMAN) is unlikely to be integrated normally in the plasma membrane (Figure S1). The fact that two predicted “low quality protein sequences” (XP_002808368.1 of *Macaca mulatta* and XP_002926069.1 of *Ailuropoda melanoleuca*) also arose by (*in silico*) fusion of the orthologous tandem genes CELR3 and S26A6 cannot be used to support gene fusion; it just illustrates the danger of error-propagation.

Analysis of the case CELR3/S26A6 ‘fusion’ thus has several important messages. First, since co-transcription of tandem genes is quite general [32], there is a significant probability that in cDNA and EST databases one will find transcripts derived from independent genes through co-transcription and alternative splicing [2,3]. If the existence of such transcripts (fusion at the transcript level) is erroneously equated with fusion at the gene level, the contribution of gene fusion to DA change will be significantly overestimated. Second, the influence of such errors in the interpretation of the data of DA evolution becomes more exaggerated as a result of error-propagation in gene predictions.

As an example of erroneous conclusion based on errors in gene prediction we may mention the case of TreeFam family TF351422 that the authors highlight as an example of a domain gain after segmental duplication and exon joining. This TreeFam family contains only primate sequences and it is claimed that after a gene duplication event one branch of the family has gained the PTEN_C2 (PF10409) domain (Additional file 8, of [29]). A closer look at the evidence presented here, however, raises doubts about the validity of the scenario proposed by the authors. As representative transcript the authors give ENST00000381866 (protein ID ENSP00000371290 equivalent with Swiss-Prot entry YM016_HUMAN). Although there is experimental support for the existence of the transcript ENST00000381866 containing the PTEN_C2 domain, the other sequences of this branch of TreeFam family TF351422 (implied to represent the ‘acceptor’ state) are very short predicted sequences whose existence is not supported by ESTs. It is noteworthy that the corresponding entries Q5T6R3_HUMAN and Q49A76_HUMAN have been deleted recently from the UniProtKB database.

As another example, we may mention the case of TreeFam family TF330855 (MSR1/SCARA5, Macrophage receptor family). In the list of ‘High confidence domain gain events’ (Table S1 of [29]) the authors claim that in this family the branch of Macrophage scavenger receptor types I and II acquired the PF03523 (Macscav_rec) domain only in mammals. As representative transcript with the gained domain they give ENST00000262101 (protein ID ENSP00000262101, equivalent with Swiss-Prot entry MSRE_HUMAN). The only non-mammalian Macrophage scavenger receptor types I and II in TreeFam TF330855 (that serves as a representative of the state before the gain of domain PF03523) is SCARA5_XENTR (ENSXETT00000037776, ENSXETP00000037776). The predicted protein ENSXETP00000037776, however, is clearly truncated at the N-terminal end (its first amino acid is an Asn) so this cannot be taken as evidence for a domain gain in mammals (Figure S2).

In summary, although one could argue that it could have been worth paying the price of small-scale analysis in order to avoid errors, a closer examination of the cases of ‘high-confidence’ DA changes indicates that the analysis of Buljan *et al.* failed to achieve this goal. First, the limited set of ‘high-confidence domain gain events’ does not properly represent the spectrum of DA changes. Second, the dataset relies on EnsEMBL therefore the analysis inherits the problems associated with errors in gene prediction (mispredicted or non-existent genes). Third, the authors rely on TreeFam where many trees contain (in addition to orthologs and paralogs) epaktologs.

## 3. Conclusions

We have shown that earlier conclusions that DA changes of Metazoan multidomain proteins occur preferentially at termini (and that the major mechanism for gains of new domains in metazoan proteins is gene fusion) are unwarranted since they reflect the fact that the databases used in these studies (e.g., UniProtKB/TrEMBL, EnsEMBL) were significantly contaminated with incomplete, abnormal and mispredicted sequences and that the authors failed to separate paralogs and epaktologs. Our studies that eliminated these problems (use of high quality Swiss-Prot sequences, separation of paralogs from epaktologs) confirmed that shuffling of mobile domains had a major role in the evolution of multidomain proteins of Metazoa and especially those formed in early vertebrates.

## Supplementary Materials

Figure S1 Alignment of the fusion product (q5y190_human) with the products of the distinct tandem genes for celr3_human and s26a6_human found on chromosome 3. Note that the fusion protein lacks a cleavable signal peptide characteristic of a type I transmembrane protein (the signal peptide of celr3_human is underlined).

Figure S2 Alignment of MSRE_HUMAN, MSRE_RABIT, MSRE_BOVIN, MSRE_MOUSE with SCARA5_XENTR. Note that the predicted sequence SCARA5_XENTR is incomplete: the N-terminal region where macrophage scavenger receptor types I and II contain a PF03523 motif is missing from this incomplete sequence.

## References

[1] Patthy L. (1991). Modular exchange principles in proteins. Curr. Opin. Struct. Biol..

[2] Akiva P., Toporik A., Edelheit S., Peretz Y., Diber A., Shemesh R., Novik A., Sorek R. (2006). Transcription-mediated gene fusion in the human genome. Genome Res..

[3] Parra G., Reymond A., Dabbouseh N., Dermitzakis E.T., Castelo R., Thomson T.M., Antonarakis S.E., Guigo R. (2006). Tandem chimerism as a means to increase protein complexity in the human genome. Genome Res..

[4] Magrangeas F., Pitiot G., Dubois S., Bragado-Nilsson E., Cherel M., Jobert S., Lebeau B., Boisteau O., Lethe B., Mallet J. (1998). Cotranscription and intergenic splicing of human galactose-1-phosphate uridylyltransferase and interleukin-11 receptor alpha-chain genes generate a fusion mRNA in normal cells. Implication for the production of multidomain proteins during evolution. J. Biol. Chem..

[5] Björklund A.K., Ekman D., Light S., Frey-Skött J., Elofsson A. (2005). Domain rearrangements in protein evolution. J. Mol. Biol..

[6] Kummerfeld S.K., Teichmann S.A. (2005). Relative rates of gene fusion and fission in multi-domain proteins. Trends Genet..

[7] Fong J.H., Geer L.Y., Panchenko A.R., Bryant S.H. (2007). Modeling the evolution of protein domain architectures using maximum parsimony. J. Mol. Biol..

[8] Behm-Ansmant I., Kashima I., Rehwinkel J., Saulière J., Wittkopp N., Izaurralde E. (2007). mRNA quality control: An ancient machinery recognizes and degrades mRNAs with nonsense codons. FEBS Lett..

[9] Patthy L. (1985). Evolution of the proteases of blood coagulation and fibrinolysis by assembly from modules. Cell.

[10] Patthy L. (1987). Intron-dependent evolution: Preferred types of exons and introns. FEBS Lett..

[11] Patthy L. (1996). Exon shuffling and other ways of module exchange. Matrix Biol..

[12] Patthy L. (1999). Genome evolution and the evolution of exon-shuffling—A review. Gene.

[13] Patthy L. (1999). Protein Evolution.

[14] Patthy L. (2003). Modular assembly of genes and the evolution of new functions. Genetica.

[15] Bányai L., Sonderegger P., Patthy L. (2010). Agrin binds BMP2, BMP4 and TGFbeta1. PLoS One.

[16] Patthy L. (2008). Alternative Splicing: Evolution. Encyclopedia of Life Sciences (ELS).

[17] Basu M.K., Poliakov E., Rogozin I.B. (2009). Domain mobility in proteins: Functional and evolutionary implications. Brief. Bioinform..

[18] Song N., Joseph J.M., Davis G.B., Durand D. (2008). Sequence similarity network reveals common ancestry of multidomain proteins. PLoS Comput. Biol..

[19] Basu M.K., Carmel L., Rogozin I.B., Koonin E.V. (2008). Evolution of protein domain promiscuity in eukaryotes. Genome Res..

[20] Chothia C., Gough J. (2009). Genomic and structural aspects of protein evolution. Biochem. J..

[21] Patthy L. (1990). Evolutionary assembly of blood coagulation proteins. Semin. Thromb. Hemost..

[22] Bernal A., Crammer K., Hatzigeorgiou A., Pereira F. (2007). Global discriminative learning for higher-accuracy computational gene prediction. PLoS Comput. Biol..

[23] Pasek S., Risler J.L., Brézellec P. (2006). Gene fusion/fission is a major contributor to evolution of multi-domain bacterial proteins. Bioinformatics.

[24] Weiner J., Beaussart F., Bornberg-Bauer E. (2006). Domain deletions and substitutions in the modular protein evolution. FEBS J..

[25] Ekman D., Björklund A.K., Elofsson A. (2007). Quantification of the elevated rate of domain rearrangements in metazoa. J. Mol. Biol..

[26] Moore A.D., Björklund A.K., Ekman D., Bornberg-Bauer E., Elofsson A. (2008). Arrangements in the modular evolution of proteins. Trends Biochem. Sci..

[27] Buljan M., Bateman A. (2009). The evolution of protein domain families. Biochem Soc Trans..

[28] Bornberg-Bauer E., Huylmans A.K., Sikosek T. (2010). How do new proteins arise?. Curr. Opin. Struct. Biol..

[29] Buljan M., Frankish A., Bateman A. (2010). Quantifying the mechanisms of domain gain in animal proteins. Genome Biol..

[30] Marsh J.A., Teichmann S.A. (2010). How do proteins gain new domains?. Genome Biol..

[31] Nagy A., Bányai L., Patthy L. (2011). Reassessing Domain Architecture Evolution of Metazoan Proteins: Major Impact of Errors Caused by Confusing Paralogs and Epaktologs. Genes.

[32] ENCODE Project Consortium (2007). Identification and analysis of functional elements in 1% of the human genome by the ENCODE pilot project. Nature.

