# Supplementary figures and images for: Reassessing Domain Architecture Evolution of Metazoan Proteins: The Contribution of Different Evolutionary Mechanisms
Source: Genes (Basel). 2011 Aug 5;2(3):578–98. doi: 10.3390/genes2030578 (PMC3927616; doi:10.3390/genes2030578)

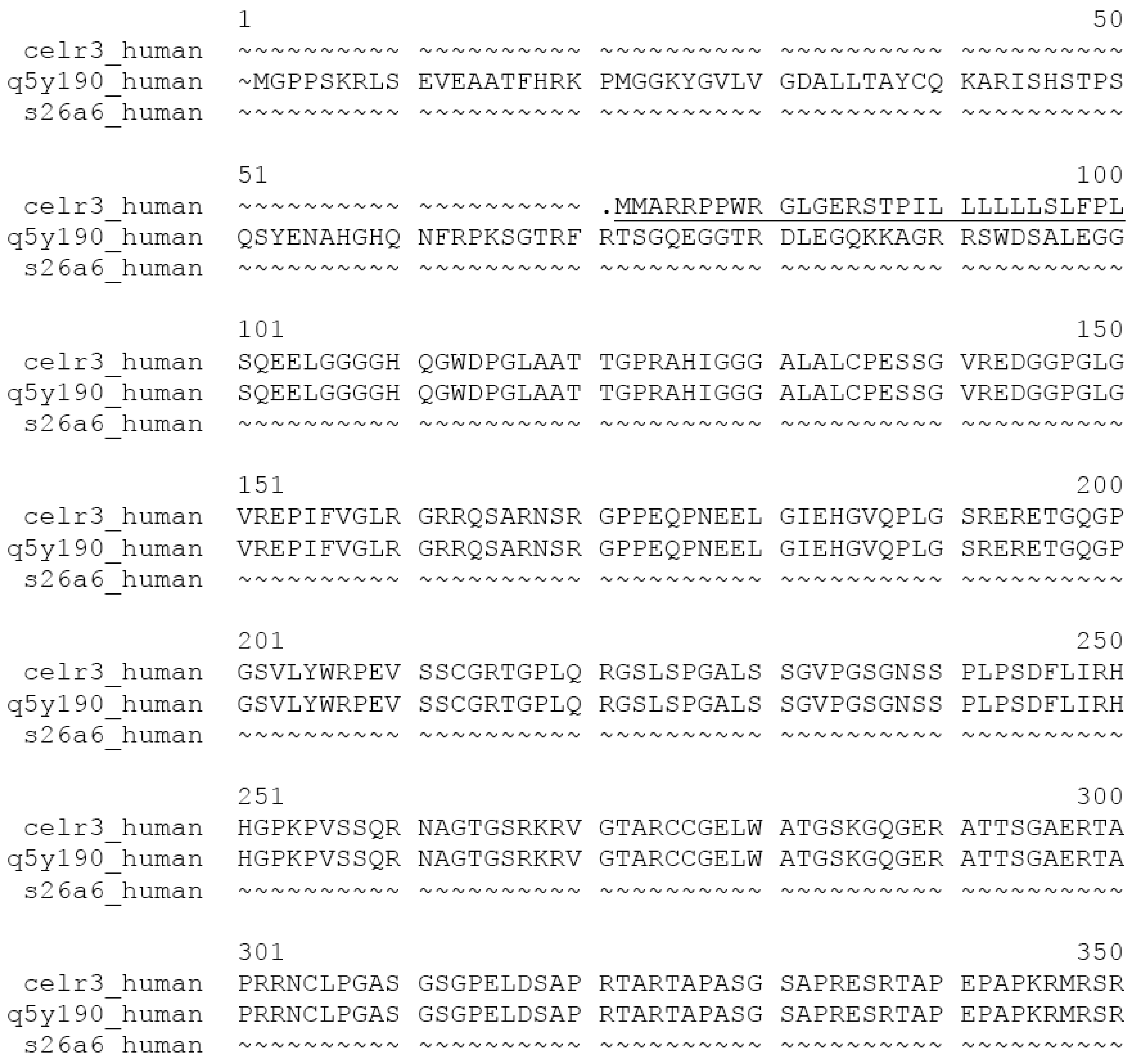

Supplement: Figure S1 — Alignment of the fusion product (q5y190_human) with the products of the distinct tandem genes for celr3_human and s26a6_human found on chromosome 3. Note that the fusion protein lacks a cleavable signal peptide characteristic of a type I transmembrane protein (the signal peptide of celr3_human is underlined). [file genes-02-00578f1a.tif]

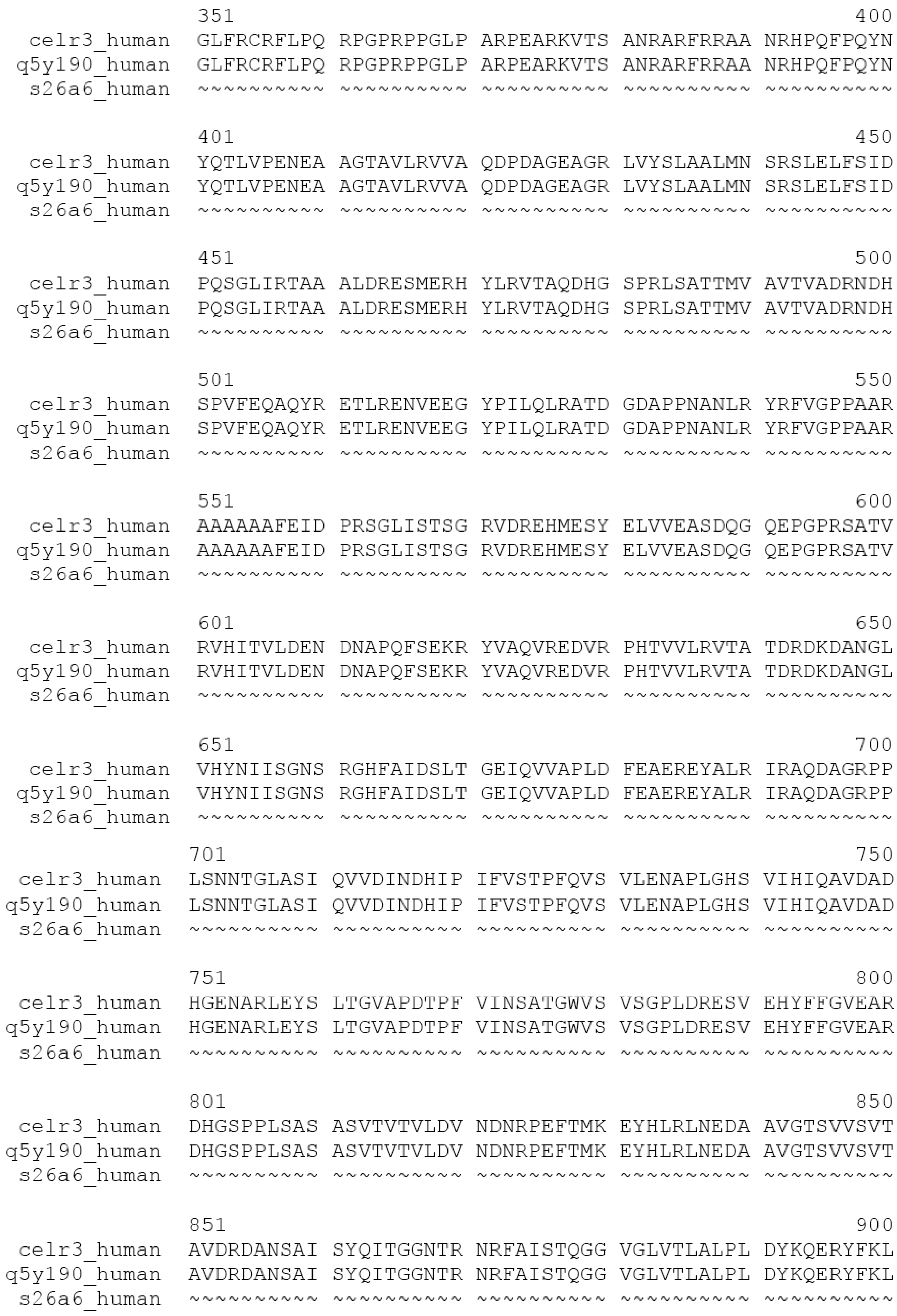

Supplement: Figure S1 — Alignment of the fusion product (q5y190_human) with the products of the distinct tandem genes for celr3_human and s26a6_human found on chromosome 3. Note that the fusion protein lacks a cleavable signal peptide characteristic of a type I transmembrane protein (the signal peptide of celr3_human is underlined). [file genes-02-00578f1b.tif]

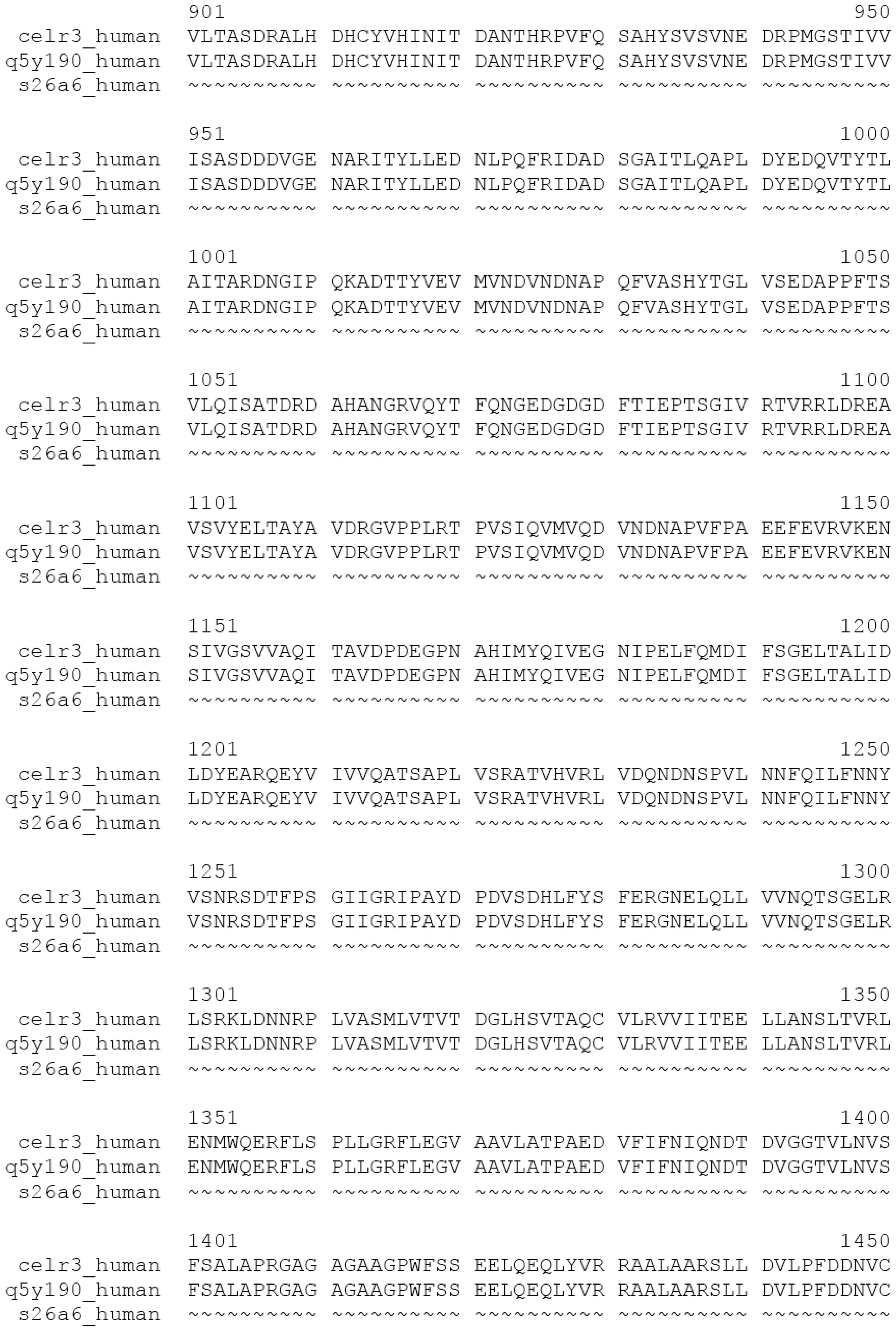

Supplement: Figure S1 — Alignment of the fusion product (q5y190_human) with the products of the distinct tandem genes for celr3_human and s26a6_human found on chromosome 3. Note that the fusion protein lacks a cleavable signal peptide characteristic of a type I transmembrane protein (the signal peptide of celr3_human is underlined). [file genes-02-00578f1c.tif]

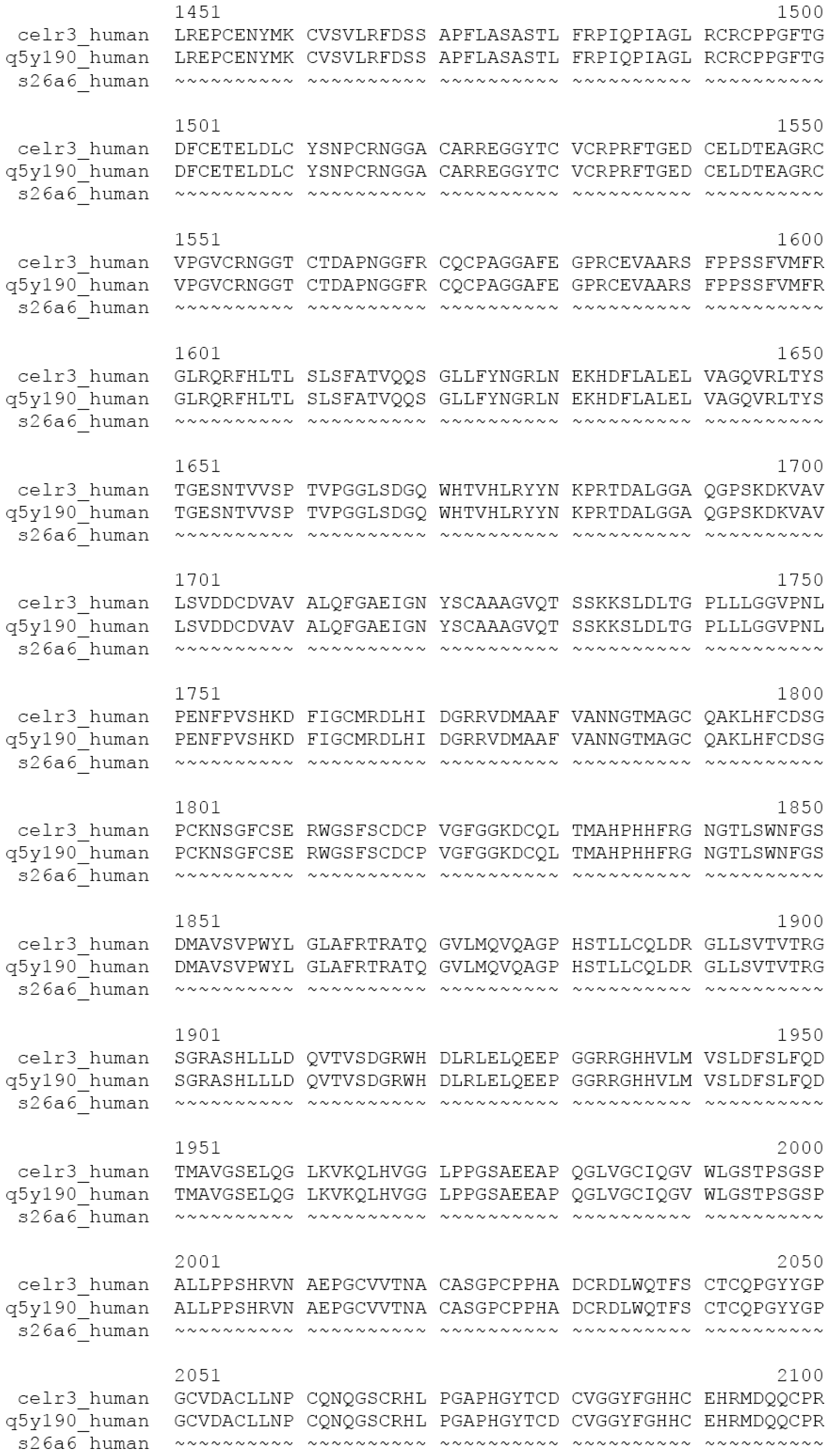

Supplement: Figure S1 — Alignment of the fusion product (q5y190_human) with the products of the distinct tandem genes for celr3_human and s26a6_human found on chromosome 3. Note that the fusion protein lacks a cleavable signal peptide characteristic of a type I transmembrane protein (the signal peptide of celr3_human is underlined). [file genes-02-00578f1d.tif]

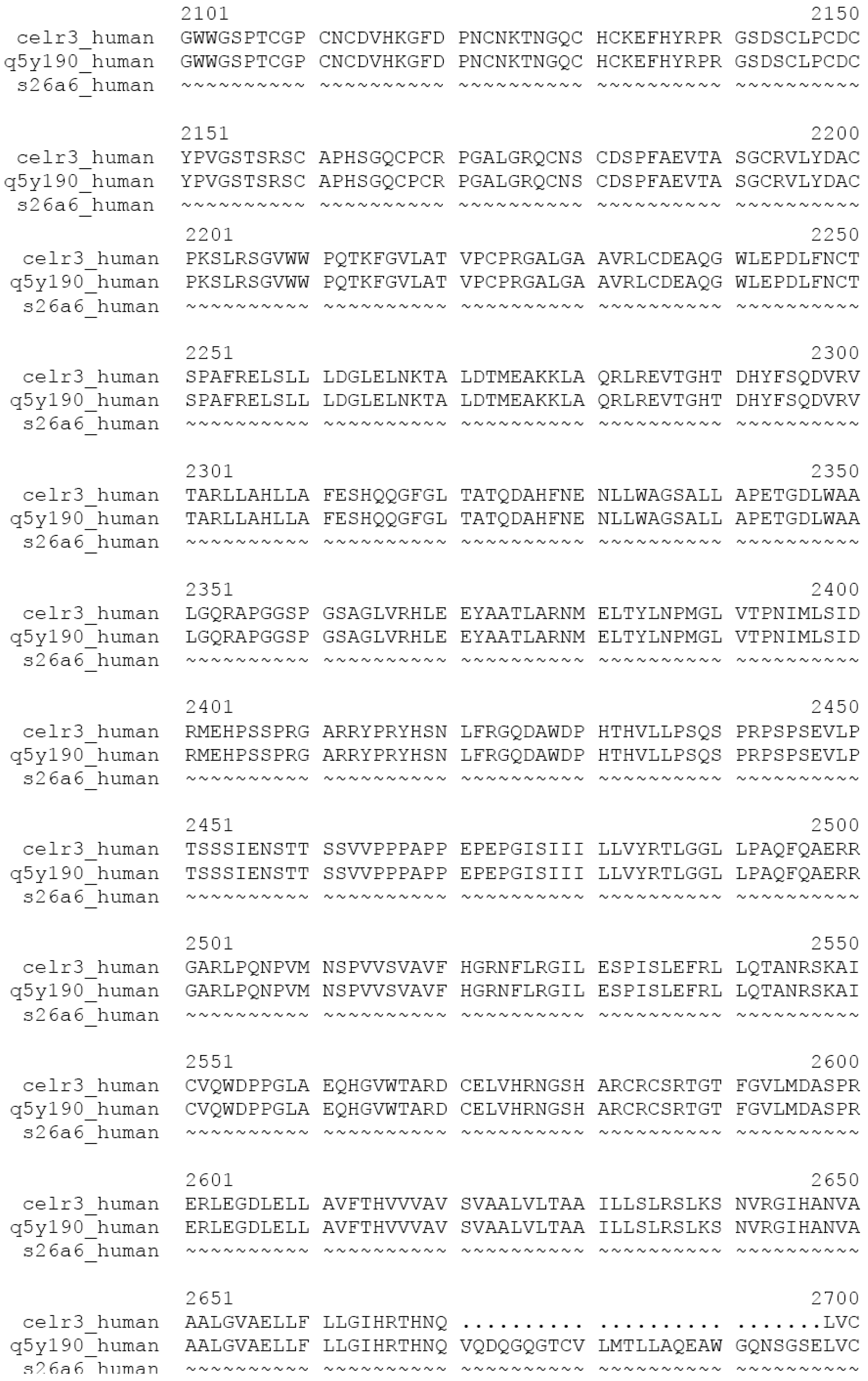

Supplement: Figure S1 — Alignment of the fusion product (q5y190_human) with the products of the distinct tandem genes for celr3_human and s26a6_human found on chromosome 3. Note that the fusion protein lacks a cleavable signal peptide characteristic of a type I transmembrane protein (the signal peptide of celr3_human is underlined). [file genes-02-00578f1e.tif]

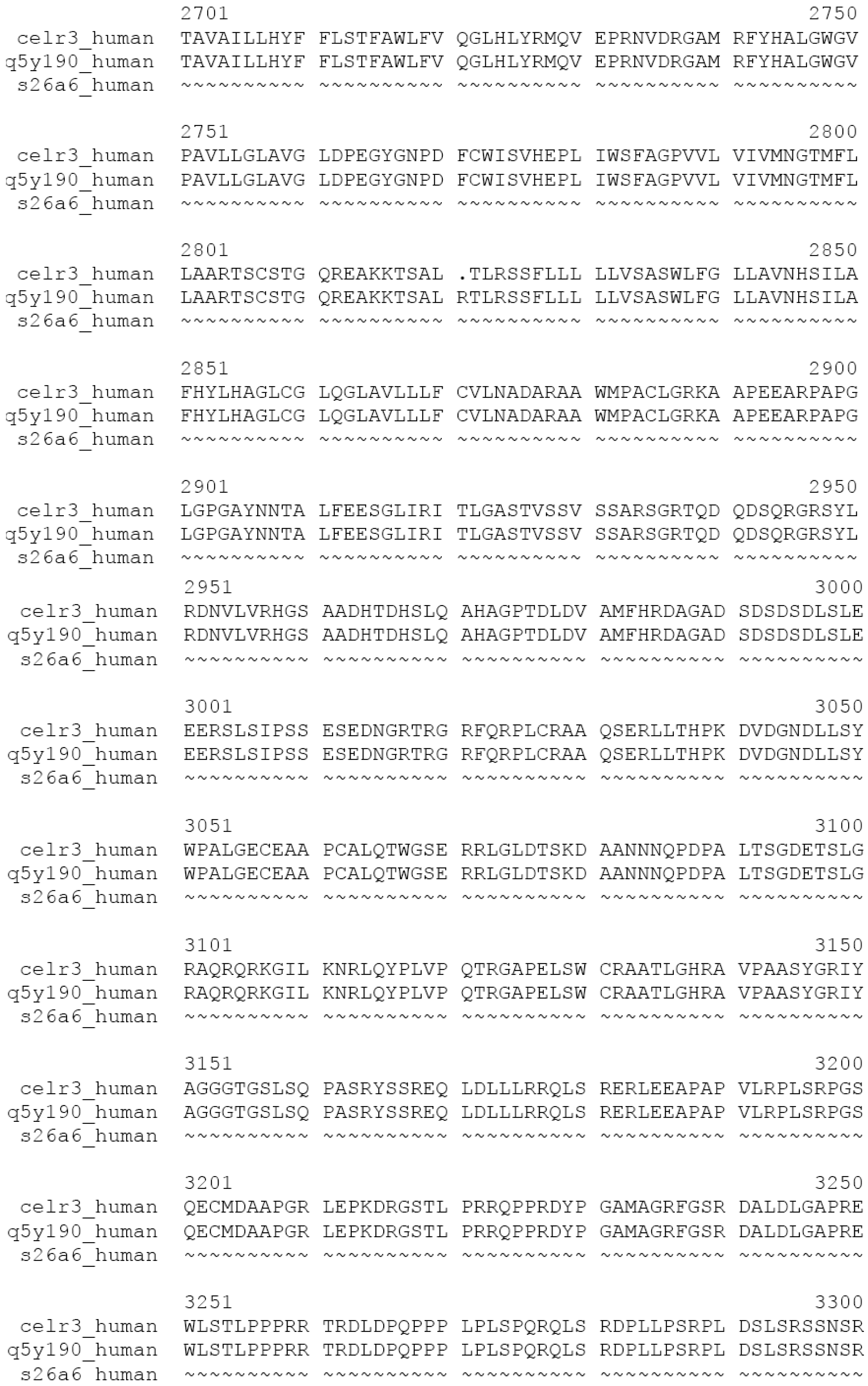

Supplement: Figure S1 — Alignment of the fusion product (q5y190_human) with the products of the distinct tandem genes for celr3_human and s26a6_human found on chromosome 3. Note that the fusion protein lacks a cleavable signal peptide characteristic of a type I transmembrane protein (the signal peptide of celr3_human is underlined). [file genes-02-00578f1f.tif]

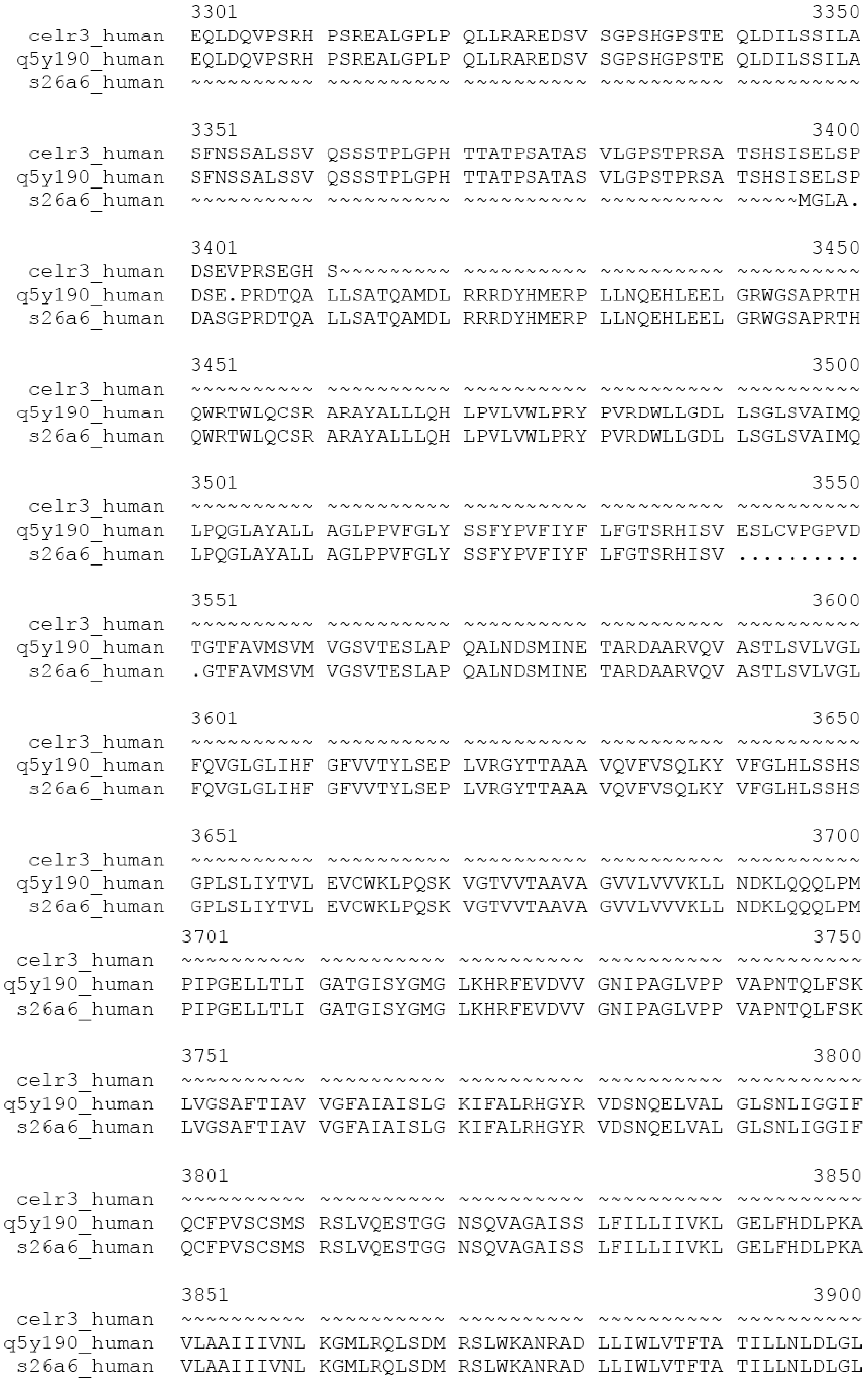

Supplement: Figure S1 — Alignment of the fusion product (q5y190_human) with the products of the distinct tandem genes for celr3_human and s26a6_human found on chromosome 3. Note that the fusion protein lacks a cleavable signal peptide characteristic of a type I transmembrane protein (the signal peptide of celr3_human is underlined). [file genes-02-00578f1g.tif]

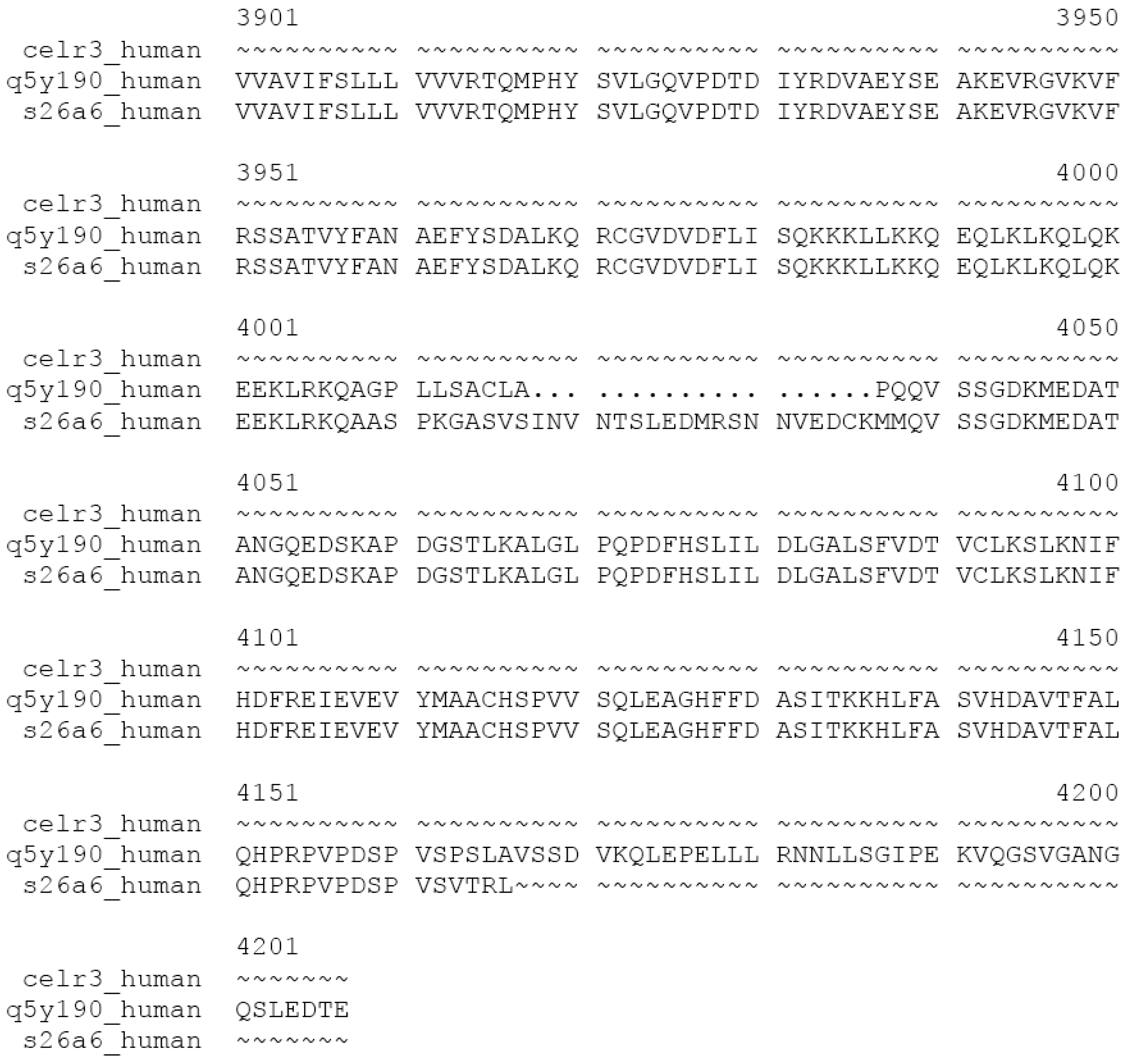

Supplement: Figure S1 — Alignment of the fusion product (q5y190_human) with the products of the distinct tandem genes for celr3_human and s26a6_human found on chromosome 3. Note that the fusion protein lacks a cleavable signal peptide characteristic of a type I transmembrane protein (the signal peptide of celr3_human is underlined). [file genes-02-00578f1h.tif]

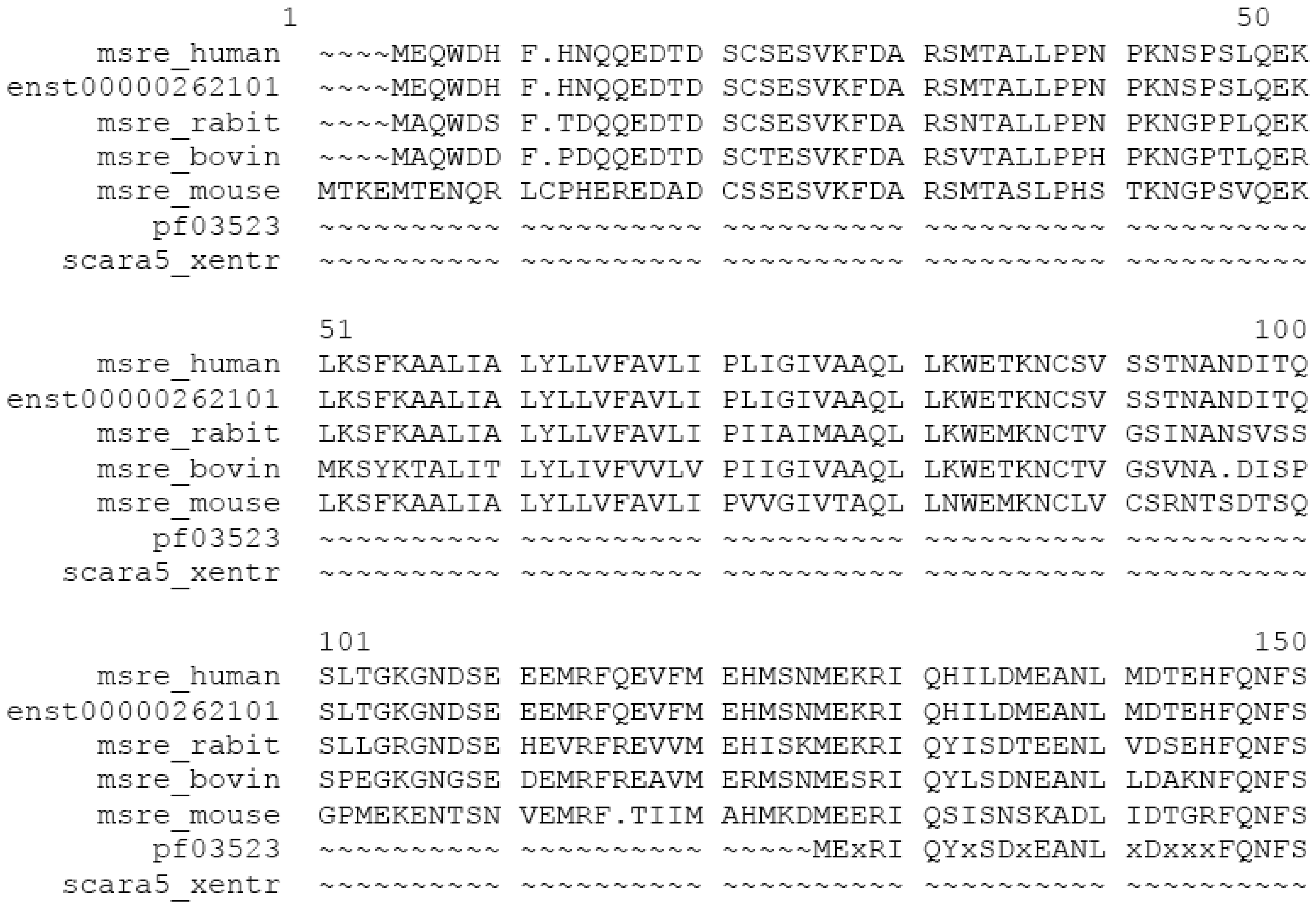

Supplement: Figure S2 — Alignment of MSRE_HUMAN, MSRE_RABIT, MSRE_BOVIN, MSRE_MOUSE with SCARA5_XENTR. Note that the predicted sequence SCARA5_XENTR is incomplete: the N-terminal region where macrophage scavenger receptor types I and II contain a PF03523 motif is missing from this incomplete sequence. [file genes-02-00578f2a.tif]

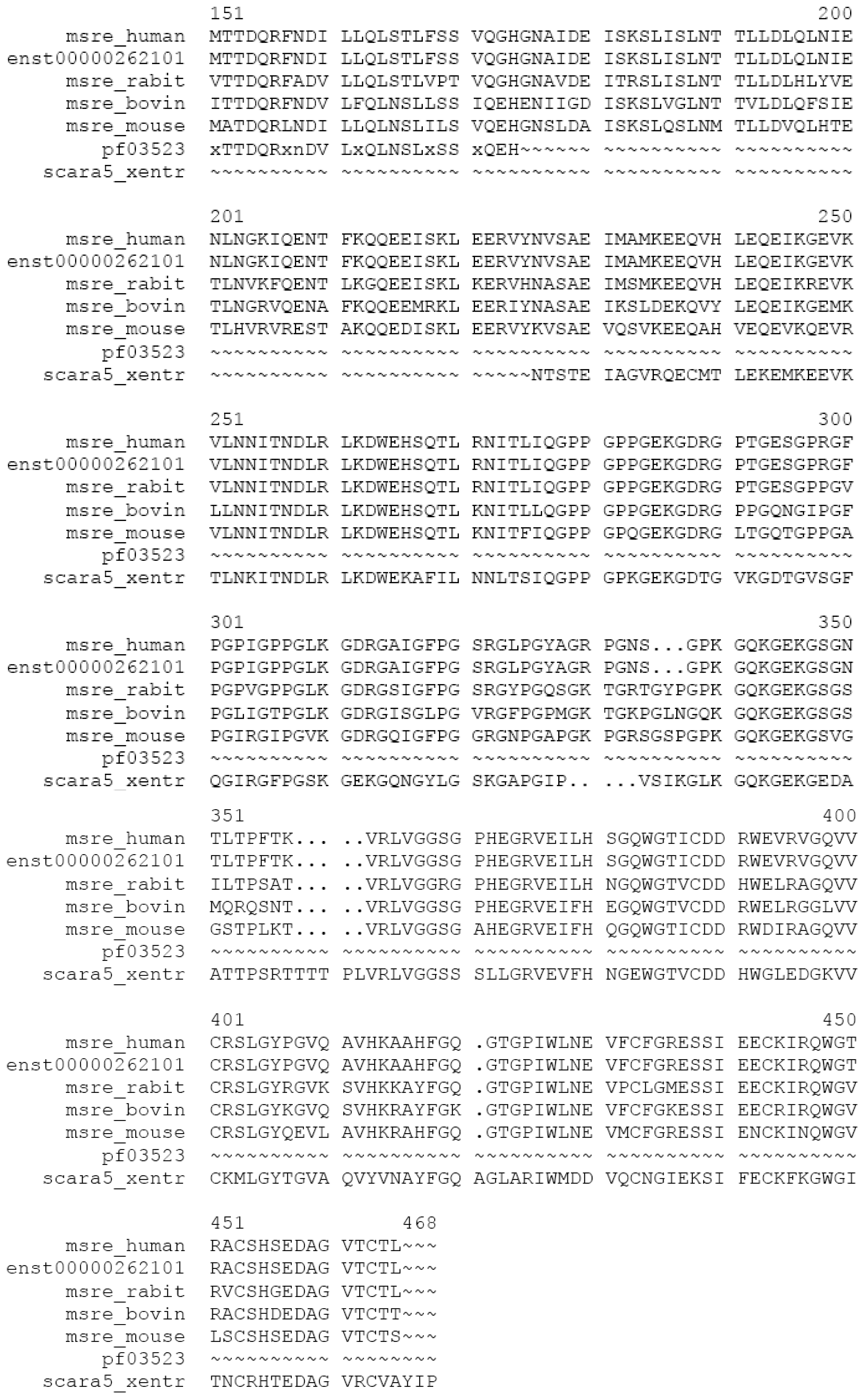

Supplement: Figure S2 — Alignment of MSRE_HUMAN, MSRE_RABIT, MSRE_BOVIN, MSRE_MOUSE with SCARA5_XENTR. Note that the predicted sequence SCARA5_XENTR is incomplete: the N-terminal region where macrophage scavenger receptor types I and II contain a PF03523 motif is missing from this incomplete sequence. [file genes-02-00578f2b.tif]
